# Simultaneous measurement of mitochondrial respiration and ATP production in tissue homogenates and calculation of effective P/O ratios

**DOI:** 10.14814/phy2.13007

**Published:** 2016-10-24

**Authors:** Karine Salin, Eugenia M. Villasevil, Sonya K. Auer, Graeme J. Anderson, Colin Selman, Neil B. Metcalfe, Christos Chinopoulos

**Affiliations:** ^1^ Institute of BiodiversityAnimal Health and Comparative MedicineUniversity of GlasgowGlasgowUK; ^2^ Department of Medical BiochemistrySemmelweis UniversityBudapestHungary; ^3^ MTA‐SE Lendület Neurobiochemistry Research GroupBudapestHungary

**Keywords:** ATPase, fluorescence, magnesium green, oxidative phosphorylation, oxygraph

## Abstract

The use of tissue homogenate has greatly aided the study of the functioning of mitochondria. However, the amount of ATP produced per oxygen molecule consumed, that is, the effective P/O ratio, has never been measured directly in tissue homogenate. Here we combine and refine existing methods previously used in permeabilized cells and isolated mitochondria to simultaneously measure mitochondrial ATP production (*J*ATP) and oxygen consumption (*J*O_2_) in tissue homogenate. A major improvement over existing methods is in the control of ATPases that otherwise interfere with the ATP assay: our modified technique facilitates simultaneous measurement of the rates of “uncorrected” ATP synthesis and of ATP hydrolysis, thus minimizing the amount of tissue and time needed. Finally, we develop a novel method of calculating effective P/O ratios which corrects measurements of *J*ATP and *J*O_2_ for rates of nonmitochondrial ATP hydrolysis and respiration, respectively. Measurements of *J*ATP and *J*O_2_ in liver homogenates from brown trout (*Salmo trutta*) were highly reproducible, although activity declined once homogenates were 2 h old. We compared mitochondrial properties from fed and food‐deprived animals to demonstrate that the method can detect mitochondrial flexibility in P/O ratios in response to nutritional state. This method simplifies studies examining the mitochondrial bioenergetics of tissue homogenates, obviating the need for differential centrifugation or chemical permeabilization and avoiding the use of nonmitochondrial ATPase inhibitors. We conclude that our approach for characterizing effective P/O ratio opens up new possibilities in the study of mitochondrial function in very small samples, where the use of other methods is limited.

## Introduction

Mitochondria are responsible for oxidation of food molecules to convert energy in the form of ATP that fuels cell functions (i.e., muscle contraction, protein synthesis, gene replication, ion homeostasis). As mitochondria produce about 90% of the cellular ATP (Lehninger et al. 1993), variation in the amount of ATP produced per unit of oxygen consumed, that is, the effective P/O ratio, have significant consequences at all levels of biological organization. Variation in effective P/O ratio is known to affect energy homeostasis in cells, and thus tissues function (Pham et al. 2014; Layec et al. 2015) and ultimately, disease pathologies and normal aging (Brand 2005; Wu et al. 2007; Kadenbach et al. 2013; Conley 2016). In extreme environmental conditions such as cold, starvation and hypoxia, mitochondrial efficiency has been suggested to be a major physiological adaptation for regulating energy homeostasis in the organism (Gnaiger et al. 2000; Trzcionka et al. 2008; Monternier et al. 2014). However, while methods of evaluating mitochondrial efficiency have received much attention (Hinkle 2005; Van Bergen et al. 2014), measuring P/O ratios has proved to be challenging (Ferguson 2010).

The mitochondrial preparation required for assaying bioenergetics involves numerous steps encompassing differential centrifugation or bath transfer (Van Bergen et al. 2014), making it technically challenging to obtain a sufficient amount of mitochondria from tissues smaller than those of a mouse (Chinopoulos et al. 2011). This limitation has motivated current efforts to implement the high yield technique of homogenization that is capable of recovering 100% of the tissue sample for use in the mitochondrial analysis (Pecinova et al. 2011; Makrecka‐Kuka et al. 2015; Rovenko et al. 2015; Ziak et al. 2015; Salin et al. 2016). Tissue homogenate offers the advantage of studying mitochondrial function close to physiological conditions, therefore differences in mitochondrial properties observed in homogenates may better reflect physiological processes that will occur in the animal (Kuznetsov et al. 2002; Burtscher et al. 2015). However, the problem emerges that homogenates contain ATPases that interfere with the ATP assay.

Techniques used to measure ATP production (*J*ATP) that are compatible with simultaneous measurement of mitochondrial oxygen consumption (*J*O_2_) have included: (1) estimation of mitochondrial ADP consumption (Estabrook 1967), (2a) direct and (2b) indirect assays of changes in ATP concentration (Ouhabi et al. 1998; Salin et al. 2010a, respectively), and (3) measurement of changes in free magnesium concentration, [Mg^2+^] (Chinopoulos et al. 2009). Technique 1 can produce biased measures of *J*ATP when using tissue homogenates due to the presence of nonmitochondrial ATPases. Technique 2b allows measurement of this ATPase activity so that the *J*ATP value can be corrected, but this correction requires twice the amount of tissue and time (Salin et al. 2012a). The presence of ATPase may also affect *J*ATP measurements using technique 3. Techniques 2a and 3 aim to block the action of ATPase so as to prevent ATP hydrolysis (Ouhabi et al. 1998; Chinopoulos et al. 2014), but the efficacy of inhibitors may depend on the tissue, study model or mitochondrial preparation.

Here, we describe a protocol for measuring effective P/O ratios that simultaneously quantifies both *J*ATP and *J*O_2_ in tissue homogenate, using a combination of techniques 2b and 3. As with technique 3, we use the magnesium‐sensitive fluorescent probe, Mg Green, to estimate changes in [Mg^2+^] (Szmacinski and Lakowicz 1996). Mitochondrial *J*ATP is calculated from the rate of change in [Mg^2+^] and is based on the unequal affinities of ATP and ADP for Mg^2+^ (Leyssens et al. 1996). Nonmitochondrial ATPase activity is measured as in technique 2b by inhibiting the mitochondrial ATP flux (Salin et al. 2010a), allowing correction of *J*ATP. Our modified technique facilitates simultaneous measurement of the rates of “uncorrected” ATP synthesis and of ATP hydrolysis, thus minimizing the amount of tissue and time needed. We validate the technique and calculate *J*ATP, *J*O_2_ and the effective P/O ratio, using liver homogenates from juvenile brown trout (*Salmo trutta*). We compare P/O ratios of fed and starved fish to determine whether our approach was a specific and sensitive assay to detect variation in P/O ratio in tissue homogenate. We chose liver because hepatic mitochondria are known to change their properties in response to food availability. Attempts to assess the role of mitochondria in the regulation of energy homeostasis during fasting have been addressed in mammals (Bobyleva‐Guarriero et al. 1984; Brand et al. 1993; Dumas et al. 2004), birds (Bobyleva‐Guarriero et al. 1984; Monternier et al. 2015), amphibians (Trzcionka et al. 2008) and fish (Savina et al. 2009; Bermejo‐Nogales et al. 2015). Of note, the role of the liver in the control of carbohydrate and lipid homeostasis is essential for providing substrates to others tissues during fasting (Postic et al. 2004). In fish, there is a large body of evidence illustrating adaptive changes in enzymatic activities of the oxidative phosphorylation pathway (Guderley et al. 2003; Frick et al. 2008; Bermejo‐Nogales et al. 2015), but whether their effective P/O ratio shows similar flexibility has so far not been determined.

## Methods

### Animal husbandry

Juvenile brown trout were obtained from a hatchery (Howietoun, UK) in summer 2015 and moved to the University of Glasgow. Here, the fish were kept in a communal tank and maintained under an 8 h light: 16 h dark photoperiod at 12°C and fed daily in excess (pellets EWOS, West Lothian, UK). In January 2016, twenty four fish were transferred to individual compartments of a stream tank. Because of the logistical constraints associated with assaying fresh mitochondria, transfers of the fish to individual compartments were staggered over 12 days (two fish per day). All fish were first acclimated for a week and fed daily to excess prior to the start of the food treatments. Half of the fish were randomly allocated to an ad libitum ration, while the other half was deprived of food (initial body mass: 12.14 ± 0.61 vs. 12.00 ± 0.57 g in the fasted and fed group). Fish were held on these treatments for 2 weeks. At the end of this period, all fish were fasted overnight. The following morning (mean ± 1 SE: 09:30 ± 00:01 h) a pair of fish was culled and their livers collected to determine mitochondrial parameters. Fasting led to reduction in body mass (final mass fasted group: 10.85 ± 2.14 g). At the same time, the fed trout had an increase in body weight (final mass fed group: 16.50 ± 3.80 g). All procedures were carried out under the jurisdiction of a UK Home Office project license (PPL 60/4292).

### Estimation of *K*_d_ of ATP and ADP for free magnesium (Mg^2+^)

**Figure 1:**
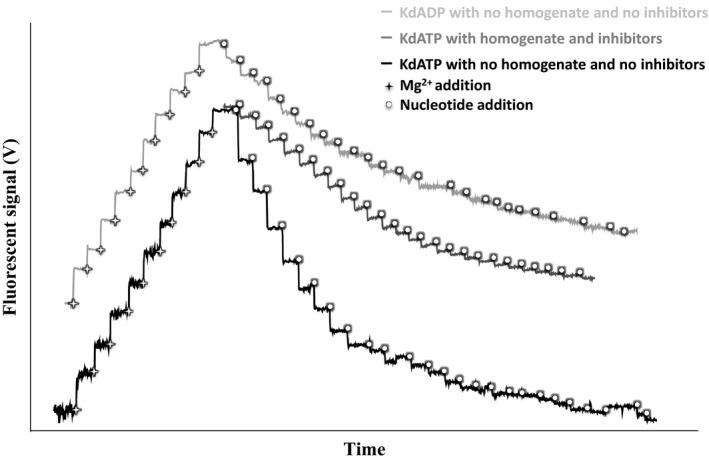
Estimation of *K*_d_ of ATP and ADP for free magnesium (Mg^2+^). Stepwise additions of MgCl_2_ (10 times 0.1 mmol/L) were performed to calibrate the relationship between the fluorescent signal and [Mg^2+^]. Stepwise additions of ATP (black line) or ADP (light gray line) were then performed to determine the affinity of those nucleotides for Mg^2+^ (25 additions of 0.2 mmol/L ATP or 0.25 mmol/L ADP). Assays were made with buffer (see main text for composition) and at 12°C, in the presence of magnesium green (2.2 *μ*mol/L), EGTA (0.1 mmol/L) and EDTA (15 *μ*mol/L), but in the absence of tissue homogenate and ATPase inhibitors. These were omitted due to the insufficient effect of ATPase inhibitors, as shown by the lack of stability of the fluorescent signal (dark gray line) after ATP additions when in the presence of homogenate and inhibitors (see main text for details).

The method for determining the affinity of the magnesium with nucleotides was adapted from Chinopoulos et al. (2014). Magnesium green fluorescence signal was detected using respirometry chambers equipped with fluorescent sensors and recorded using DatLab software (Oxygraph‐2k high resolution, Oroboros Instruments, Innsbruck Austria, as in [Chinopoulos et al. 2014]). The main adjustment of the assay of the K_*d*_ is a measurement in the absence of tissue homogenate and ATPase inhibitors (see below). Thus, assays were made in duplicate in a buffer containing 20 mmol/L Taurine, 10 mmol/L KH_2_PO_4_, 20 mmol/L HEPES, 110 mmol/L D‐sucrose, 60 mmol/L K‐lactobionate, 1 g/L BSA fatty acid free, pH 7.2 and at 12°C. The titration protocol was as follows: the buffer was supplemented with pyruvate (5 mmol/L) and malate (0.5 mmol/L). Magnesium green (2.1 *μ*mol/L), EGTA (0.1 mmol/L) and EDTA (15 *μ*mol/L) were subsequently added to the chamber. Then, stepwise additions of MgCl_2_ were performed for calibration of the fluorescent signal into Mg^2+^ (Fig. 1). Succinate (10 mmol/L) was then added. Stepwise additions of ATP or ADP were then performed to determine the affinity of those nucleotides for Mg^2+^ (25 additions of 0.2 mmol/L ATP or 0.25 mmol/L ADP) (Fig. 1). The two *K*_d_ values were determined using the least squares method, described in detail in Chinopoulos et al. (2014). The binding affinity (*K*_d_) of ATP and ADP for Mg^2+^ was calculated, using the method of Chinopoulos et al. (2014); the values were *K*_d‐ATP_ = 0.1545 mmol/L and *K*_d‐ADP_ = 2.1333 mmol/L.

The method for determining the affinity of the magnesium with nucleotides in the presence of mitochondria and ATPase inhibitors (as in Chinopoulos et al. 2014) was not used here due to the insufficient effect of ATPase inhibitors. In particular, there was a lack of stability of the fluorescent signal (Fig. 1) after ATP additions when in the presence of homogenate (5 mg wet mass liver per ml buffer) and inhibitors (25 *μ*mol/L adenylate kinase inhibitor Ap5A, 2 *μ*g/mL oligomycin, 4 *μ*mol/L carboxylatractyloside (cATR), 0.2 mmol/L beryllium sulfate tetrahydrate, 5 mmol/L sodium trifluoride and 30 *μ*mol/L sodium orthovanadate). We therefore omitted ATPase inhibitors when making measurements of *K*_d_, *J*ATP and *J*O_2_. Thus, the insufficient effect of ATPase inhibitors on liver homogenate motivates us to further develop a method to quantify the rate of ATP hydrolysis.

### Mitochondrial homogenate preparation

Liver homogenate was prepared as in (Salin et al. 2016). Briefly, tissues (mean ± 1 SE: 43.08 ± 2.02 mg) were minced to obtain a shredded solution, which was then homogenized with a Potter‐Elvehjem homogenizer in a buffer containing 20 mmol/L Taurine, 10 mmol/L KH_2_PO_4_, 20 mmol/L HEPES, 110 mmol/L D‐sucrose, 60 mmol/L K‐lactobionate, 1 g/L BSA fatty acid free, pH 7.2. Homogenates were then diluted to obtain a final concentration of 5 mg/mL. The entire procedure was carried out at 4°C and completed within 30 min of the fish being culled. A sample of liver homogenate was then immediately added to one of the two respirometry chambers. The leftover preparation of liver homogenate was preserved on ice for use in a replicate trial.

### Mitochondrial rates of oxidation and phosphorylation: measurement

Oxygen and magnesium green fluorescence signals were detected simultaneously, using respirometry chambers equipped with fluorescent sensors (as in Chinopoulos et al. 2014). Pure oxygen gas was added to the respirometry chambers to reach a concentration of 550 *μ*mol/L. The titration protocol was as follows: respiration was stimulated by adding pyruvate (5 mmol/L) and malate (0.5 mmol/L). Magnesium green (2.1 *μ*mol/L), EGTA (0.1 mmol/L) and EDTA (15 *μ*mol/L) were subsequently added to the chamber. Then, stepwise additions of MgCl_2_ were performed for calibration of the fluorescent signal into Mg^2+^. Succinate (10 mmol/L) was then added. State 3 was reached by adding a saturating concentration of ADP (2 mmol/L). In this condition, changes in oxygen and ATP concentrations in the chamber are representative of raw fluxes in oxygen and ATP (*J*O_2‐raw_ and *J*ATP_raw_, respectively). *J*O_2‐raw_ reflects the rate of oxygen consumption by the mitochondria but also by nonmitochondrial reactions; *J*ATP_raw_ is the balance between the rate of mitochondrial ATP production and the rate of ATP disappearance due to the activity of the various ATPase enzymes (phosphorylases, phosphatases and kinases; for further details see Chinopoulos et al. 2014). Addition of cATR (4 *μ*mol/L), an inhibitor of ATP‐ADP exchanger, allowed calculation of the rate of ATP disappearance due to ATPase activity under state 4 conditions (*J*ATP_St4_). Addition of complex I inhibitor, rotenone (0.5 *μ*mol/L) and complex III inhibitor, antimycin A (2.5 *μ*mol/L) determined nonmitochondrial, or residual, oxygen consumption (*J*O_2‐ROX_). At each step the rates of changes in ATP and/or oxygen were allowed to stabilize. An identical trial was run on the homogenate obtained from the same fish 2 h later, in order to determine the repeatability of measurements and the stability of measurements in relation to time since sample preparation.

### Analysis of mitochondrial rates of oxidation and phosphorylation

**Figure 2:**
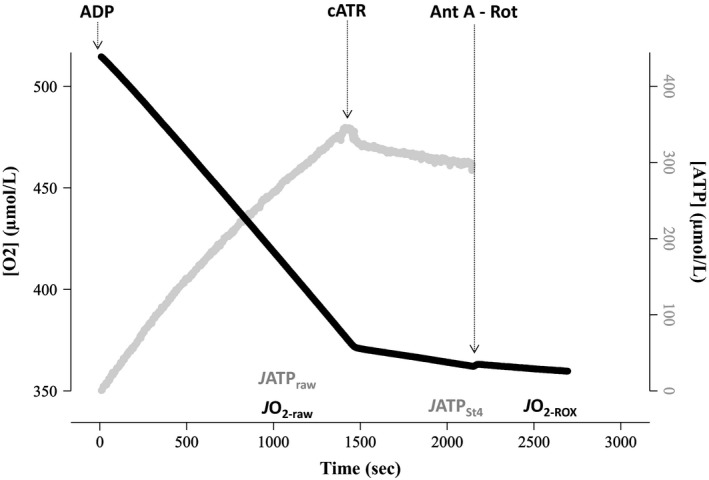
Output from a representative experiment, using homogenized brown trout liver, showing changes in oxygen and ATP content as a function of time. The substrates pyruvate, malate and succinate, as well as free magnesium, were present at the start of the experiment. State 3 respiration was initiated by adding 2 mmol/L ADP; the disappearance of oxygen (black line) was matched by the appearance of ATP (gray line). State 3 parameters stabilized 15 min after adding ADP, at which point rates of oxygen consumption (*J*O_2‐raw_) and ATP production (*J*ATP_raw_) were measured for a further 5 min. The rate of ATP disappearance due to the activity of ATPase (*J*ATP_S__t4_) was quantified by inhibiting the mitochondrial ATP‐ADP exchange with carboxyatractyloside (cATR) (note the cessation of ATP production at this point, and reduction in oxygen consumption). Finally, the nonmitochondrial oxygen consumption (*J*O_2‐_ _ROX_) was estimated by inhibiting the respiratory chain with antimycin A (Ant A) and rotenone (Rot). Mitochondrial parameters were measured at 5 mg wet mass liver per ml at 12°C; Oxygen content in the respirometry chambers never dropped below 300 *μ*mol/L, so maintaining the liver above sensitive levels (≈100 *μ*mol/L in our conditions, data not shown).

Mitochondrial oxidation and phosphorylation were analyzed with Microsoft Excel 2010. Free Mg^2+^ was converted into extra‐mitochondrial ATP concentration, using standard binding equations previously described (Chinopoulos et al. 2014). The rate of ATP production was calculated from the linear regression of ATP concentration as a function of time (Fig. 2); *J*ATP_raw_ was averaged over a 5 min period, starting 15 min post‐ADP addition while *J*ATP_St4_ was averaged over a 5 min period after flux stabilisation. *J*ATP_St4_ was then subtracted from *J*ATP_raw_ to obtain the state 3 rate of mitochondrial ATP production (*J*ATP_St3_), which was expressed in pmol ATP/sec/mg wet weight of liver. Changes in oxygen concentration over time were calculated in a similar way (Fig. 2), *J*O_2‐raw_ being averaged over 5 min, starting 15 min post‐ADP addition, while *J*O_2‐ROX_ was averaged over 1–2 min after flux stabilisation. *J*O_2‐ROX_ was then subtracted from *J*O_2‐raw_ to obtain the state 3 respiration rate (*J*O_2‐St3_), expressed in pmol O_2_/sec/mg wet weight of liver. Finally, the P/O ratio was calculated as the ratio of *J*ATP_St3_ to twofold *J*O_2‐St3_; the rate of oxygen consumption is doubled since each molecule of oxygen is comprised of two oxygen atoms.

### Chemicals

Magnesium green was purchased from Thermo Fisher Scientific (Renfrew, UK), ADP was purchase from Merck (Hoddesdon, UK), water was purchased from VWR (Lutterworth, UK). All other chemicals were purchased from Sigma‐Aldrich (Dorset, UK).

### Statistical analysis

The reproducibility of the estimates of *J*O_2‐St3_, *J*ATP_St3_, and the P/O ratio of the two trials from the same homogenate was assessed, using Intraclass Correlation Coefficients (ICC). Paired *t*‐tests were then used to test for any consistent shifts in the values of the measurements between the first and second trial (2 h later). Finally, linear mixed models were employed to test whether fed and fasted fish differed significantly in their hepatic mitochondrial properties (mean value of *J*O_2‐St3_, *J*ATP_St3,_ and P/O ratio) while taking into account any effect of testing date (included as a random factor). All statistical analyses were performed in SPSS Statistics 21 (Chicago, IL).

## Results and Discussion

**Figure 3:**
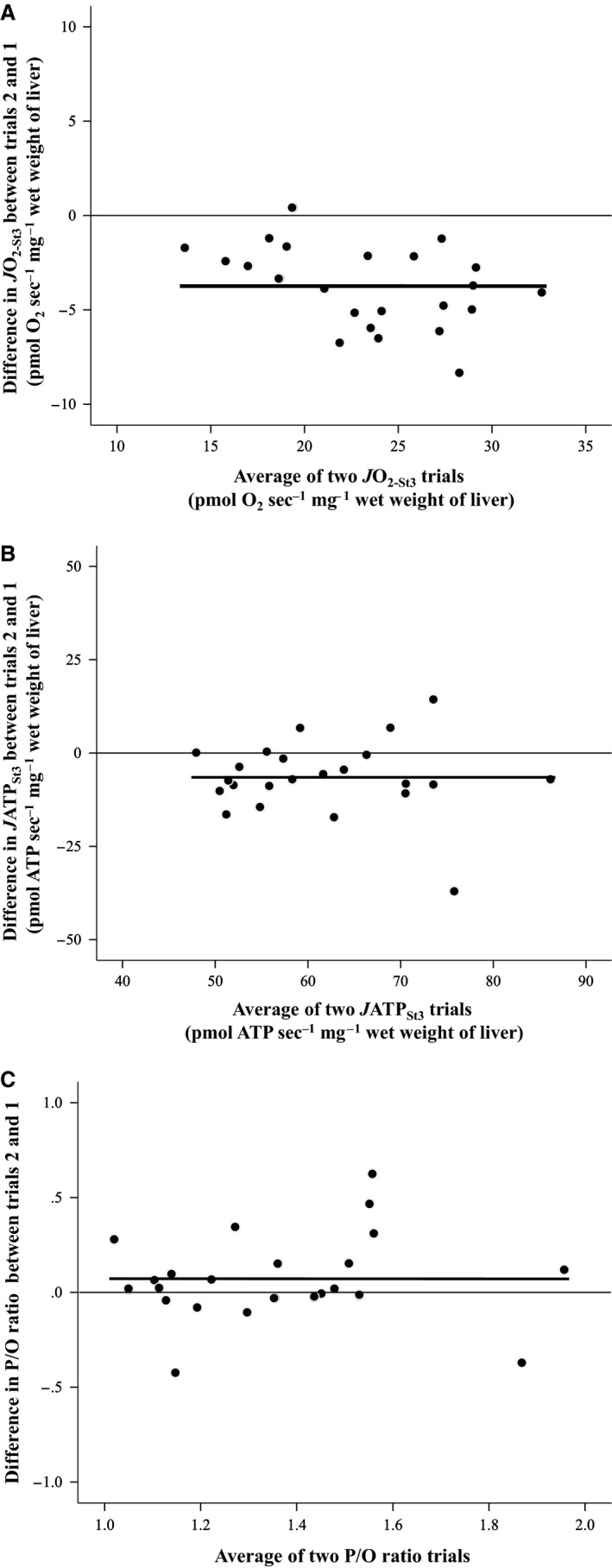
Changes in mitochondrial properties as a result of time since liver homogenization, shown as the difference between measurements made immediately versus 2 h after homogenization, and plotted against the mean of the two values. (A) Hepatic mitochondrial oxygen consumption *J*O_2‐St3_ (drift between trials: paired *t* = 8.322, *P *<* *0.001). (B) Liver mitochondrial ATP production *J*ATP_S__t3_ (drift between trials: paired *t* = 3.099, *P = *0.005). (C) Liver mitochondrial estimated P/O ratio (drift between trials: paired *t* = −1.476, *P *=* *0.154). *N* = 23 fish in all cases. Thick lines represent the mean value of the difference, and the thin line a difference of zero (i.e., no difference in measurement between the two trials).

The reproducibility of *J*O_2‐St3_ and *J*ATP_St3_ measurements between the two replicate trials from the same liver homogenate was high (*J*O_2‐St3_: ICC *r *=* *0.953, df = 22, *P *<* *0.001; *J*ATP_St3_: ICC *r *=* *0.746, df = 22, *P *=* *0.001). As a result, the P/O ratio was also highly reproducible (ICC *r *=* *0.778, df = 22, *P *<* *0.001). However, the mitochondrial activity of homogenates declined over time, as indicated by significantly lower *J*O_2‐St3_ and *J*ATP_St3_ measurements in the 2nd assay that was conducted 2 h after the first (Fig. 3A and B). This led to a tendency for the P/O ratio to also change over time, although this change was not significant (Fig. 3C). This lack of a significant change in P/O ratio with time posthomogenization was due to both the numerator and denominator changing in the same direction and at broadly similar rates, but this may not be the case in other tissues or for greater time intervals between assays. The temporal stability of mitochondrial properties should therefore always be assessed when using this method, and delays between sample preparation and measurement kept to a minimum.

**Figure 4:**
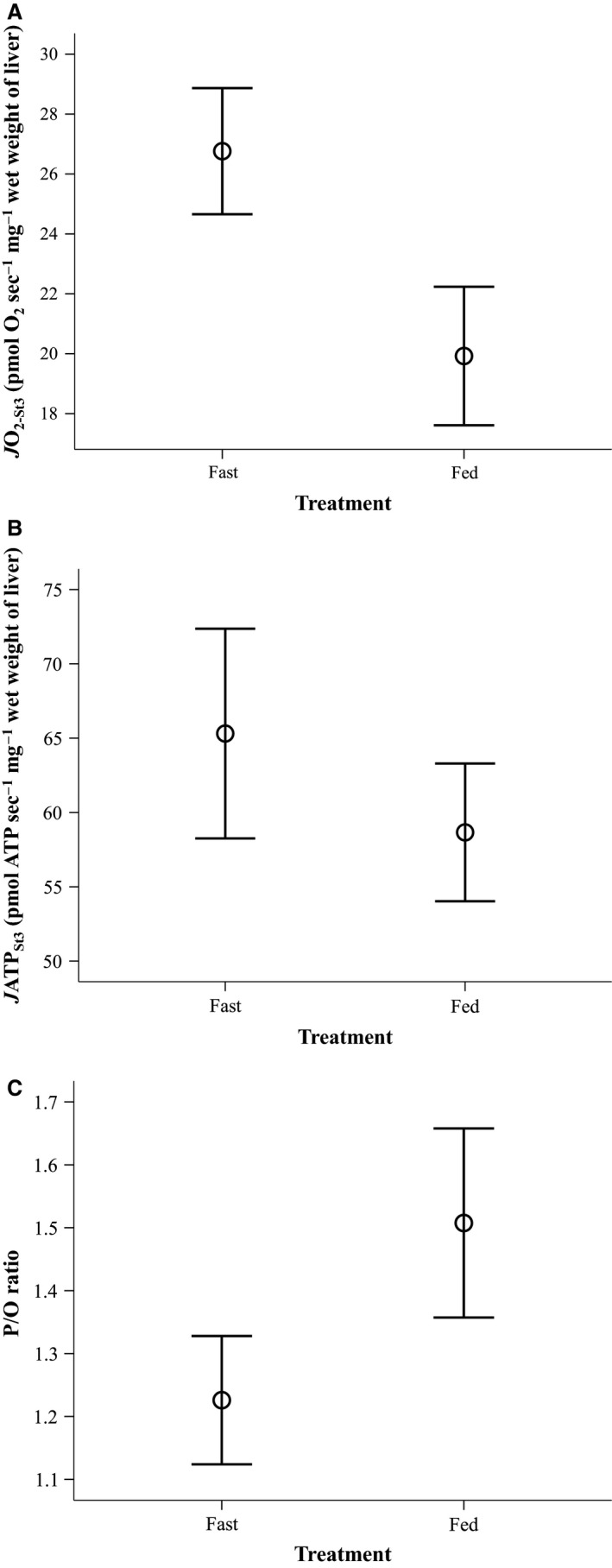
Liver mitochondrial ATP production and oxygen consumption values of fasted and ad libitum‐fed groups of brown trout. Food treatment significantly affected (A) mitochondrial oxygen consumption (*J*O_2‐St3_: *F*_1,11_ = 28.677, *P* < 0.001), (B) mitochondrial ATP production to a lower extent (*J*ATP_S__t3_: *F*_1,11_ = 4.805, *P* = 0.051) and (C) mitochondrial P/O ratio (*F*_1,11_ = 20.937, *P* = 0.001). Shown are average values from the two replicate measurements of mitochondrial properties per fish. Data are plotted as mean ± 1 SE. *N* = 12 fish per treatment group.

The liver mitochondria of trout starved for 14 days showed significant differences in their properties compared to those of fed conspecifics (Fig. 4). The mitochondria from fasted fish exhibited significantly higher rates of oxygen consumption and a trend for an increase in rates of ATP production in comparison with those of fed fish (Fig. 4A and B). As a result of this substantial increase in *J*O_2_ but lesser increase in *J*ATP, the P/O ratio of liver mitochondria from food‐deprived fish was significantly reduced compared to that of fed fish (Fig. 4C). While an increase in coupling efficiency has been suggested to be a primary means by which animals reduce energy expenditure when faced with harsh conditions (Theron et al. 2000; Monternier et al. 2014; Salin et al. 2015), other studies have proposed that the P/O ratio is a major constraint in optimizing the efficiency of energy utilization (Konstantinov et al. 1976; Bobyleva‐Guarriero et al. 1984; Abele et al. 2002; Bottje and Carstens 2009; Salin et al. 2012b; Conley et al. 2013). The effect of starvation on mitochondrial properties is also likely to depend on both the tissue (Trzcionka et al. 2008; Brown et al. 2012; Crescenzo et al. 2012) and the animal species involved (Emel'yanova et al. 2007; Bottje and Carstens 2009). A reduction in the apparent P/O ratio in the liver of the food‐deprived fish is consistent with previous studies of chickens (Bobyleva‐Guarriero et al. 1984), but contrasts with results found in the skeletal muscle of starving penguins (Monternier et al. 2014) and in the livers of starved rats (Jung and Henke 1997). The degree to which mitochondrial properties change may also depend on the severity of the energetic challenge, for example, the duration of food deprivation (Hung et al. 1997; Salin et al. 2010b). It would be profitable to test whether the effect of fasting on mitochondrial coupling efficiency is consistent across different tissues from the same animal, and whether the direction and magnitude of change in P/O ratio is dependent on the duration of food deprivation.

### Perspectives and significance

The aim of this study was to measure simultaneously *J*ATP and *J*O_2_ and hence the P/O ratio in tissue homogenate. Our approach was to use the magnesium‐sensitive fluorescent probe, Mg Green, to estimate changes in [Mg^2+^] and in turn *J*ATP, while correcting for ATPase interference. In our study the average P/O ratio for pyruvate, malate and succinate oxidation was 1.367, which is only slightly lower than maximal theoretical values reported for pyruvate and malate oxidation (2.727) and for oxidation of succinate (1.636) (Brand 2005; Watt et al. 2010). This supports our conclusion that the method is appropriate for measuring effective P/O ratios. In the conditions pertaining in our experiment, inhibitors for ATPase failed to fully inhibit ATP hydrolysis (Fig. 1). This limitation has been overcome by inhibiting the ATP‐ADP exchanger with a highly specific inhibitor, the cATR, in order to quantify rates of ATP hydrolysis. This extra step of the protocol does not require any more tissue and only a minimal amount of time for the mitochondria to reach a steady state 4. Our method can even be used to analyze P/O ratios from permeabilized cells and isolated mitochondria. Thus, P/O ratio comparisons among species, individuals and tissues can be carried out, even across a range of mitochondrial preparations.

The Mg Green method has previously enabled evaluation of ATP production in permeabilized cells and isolated mitochondria (Chinopoulos et al. 2009, 2014). However, such samples are not always appropriate in studies of the energy metabolism of an organism. Moreover, ecology and evolutionary biology requires methods for measuring mitochondrial traits that can be applied to nonmodel organisms that potentially exhibit smaller tissues and a lower mitochondrial density than a laboratory mouse. We have for the first time demonstrated the feasibility of a method for measuring mitochondrial coupling efficiencies on homogenized tissue, showing that our method can be used to calculate effective P/O ratios from tissue samples as small as tens of milligrams. This protocol may facilitate the study of P/O ratios in further models, and shed light on the role of mitochondrial coupling efficiency in influencing fitness‐related traits.

## Conflict of Interest

The authors declare they have no conflict of interest.

## References

[phy213007-bib-0001] Abele, D. , K. Heise , H. O. Pörtner , and S. Puntarulo . 2002 Temperature‐dependence of mitochondrial function and production of reactive oxygen species in the intertidal mud clam *Mya arenaria* . J. Exp. Biol. 205:1831–1841. 12077159 10.1242/jeb.205.13.1831

[phy213007-bib-0002] Bermejo‐Nogales, A. , J. A. Calduch‐Giner , and J. Pérez‐Sánchez . 2015 Unraveling the molecular signatures of oxidative phosphorylation to cope with the nutritionally changing metabolic capabilities of liver and muscle tissues in farmed fish. PLoS ONE 10:e0122889. 25875231 10.1371/journal.pone.0122889 PMC4398389

[phy213007-bib-0003] Bobyleva‐Guarriero, V. , P. E. Hughes , I. Ronchetti‐Pasquali , and H. A. Lardy . 1984 The influence of fasting on chicken liver metabolites, enzymes and mitochondrial respiration. Comp. Biochem. Physiol. B 78:627–632. 6478793 10.1016/0305-0491(84)90109-3

[phy213007-bib-0004] Bottje, W. G. , and G. E. Carstens . 2009 Association of mitochondrial function and feed efficiency in poultry and livestock species. J. Anim. Sci. 87:E48–E63. 19028862 10.2527/jas.2008-1379

[phy213007-bib-0005] Brand, M. D. 2005 The efficiency and plasticity of mitochondrial energy transduction. Biochem. Soc. Trans. 33:897–904. 16246006 10.1042/BST0330897

[phy213007-bib-0006] Brand, M. D. , M. E. Harper , and H. C. Taylor . 1993 Control of the effective P/O ratio of oxidative phosphorylation in liver mitochondria and hepatocytes. Biochem. J. 291:739–748. 8489502 10.1042/bj2910739 PMC1132431

[phy213007-bib-0007] Brown, J. C. L. , D. J. Chung , K. R. Belgrave , and J. F. Staples . 2012 Mitochondrial metabolic suppression and reactive oxygen species production in liver and skeletal muscle of hibernating thirteen‐lined ground squirrels. Am. J. Physiol. Regul. Integr. Comp. Physiol. 302:R15–R28. 21993528 10.1152/ajpregu.00230.2011

[phy213007-bib-0008] Burtscher, J. , L. Zangrandi , C. Schwarzer , and E. Gnaiger . 2015 Differences in mitochondrial function in homogenated samples from healthy and epileptic specific brain tissues revealed by high‐resolution respirometry. Mitochondrion 25:104–112. 26516105 10.1016/j.mito.2015.10.007

[phy213007-bib-0009] Chinopoulos, C. , S. Vajda , L. Csanády , M. Mándi , K. Mathe , and V. Adam‐Vizi . 2009 A novel kinetic assay of mitochondrial ATP‐ADP exchange rate mediated by the ANT. Biophys. J. 96:2490–2504. 19289073 10.1016/j.bpj.2008.12.3915 PMC2907717

[phy213007-bib-0010] Chinopoulos, C. , S. F. Zhang , B. Thomas , V. Ten , and A. A. Starkov . 2011 Isolation and functional assessment of mitochondria from small amounts of mouse brain tissue Pp. 311–324, Vol. 793 *in* Manfredi G. and Kawamata H. eds. Neurodegeneration: methods and protocols. Humana Press Inc, Totowa, NJ. 10.1007/978-1-61779-328-8_20 PMC3627350 21913109

[phy213007-bib-0011] Chinopoulos, C. , G. Kiss , H. Kawamata , and A. A. Starkov . 2014 Chapter seventeen – measurement of ADP‐ATP exchange in relation to mitochondrial transmembrane potential and oxygen consumption Pp. 333–348, Vol. 542 *in* Lorenzo G. and Guido K., eds. Methods in enzymology. Academic Press, San Diego. 10.1016/B978-0-12-416618-9.00017-0 PMC4630003 24862274

[phy213007-bib-0012] Conley, K. E. 2016 Mitochondria to motion: optimizing oxidative phosphorylation to improve exercise performance. J. Exp. Biol. 219:243–249. 26792336 10.1242/jeb.126623 PMC6514472

[phy213007-bib-0013] Conley, K. E. , S. A. Jubrias , M. E. Cress , and P. Esselman . 2013 Exercise efficiency is reduced by mitochondrial uncoupling in the elderly. Exp. Physiol. 98:768–777. 23085769 10.1113/expphysiol.2012.067314

[phy213007-bib-0014] Crescenzo, R. , F. Bianco , I. Falcone , P. Coppola , A. G. Dulloo , G. Liverini , et al. 2012 Mitochondrial energetics in liver and skeletal muscle after energy restriction in young rats. Br. J. Nutr. 108:655–665. 22085624 10.1017/S0007114511005903

[phy213007-bib-0015] Dumas, J. , D. Roussel , G. Simard , O. Douay , F. Foussard , Y. Malthiery , et al. 2004 Food restriction affects energy metabolism in rat liver mitochondria. Biochim. Biophys. Acta 1670:126–131. 14738995 10.1016/j.bbagen.2003.11.002

[phy213007-bib-0016] Emel'yanova, L. V. , M. V. Savina , E. A. Belyaeva , and I. V. Brailovskaya . 2007 Peculiarities of functioning of liver mitochondria of the river lamprey *Lampetra fluviatilis* and the common frog *Rana temporaria* at periods of suppression and activation of energy metabolism. J. Evol. Biochem. Physiol. 43:564–572. 18265558

[phy213007-bib-0017] Estabrook, R. W. 1967 Mitochondrial respiratory control and the polarographic measurement of ADP:O ratios. Methods Enzymol. 10:41–47.

[phy213007-bib-0018] Ferguson, S. J. 2010 ATP synthase: from sequence to ring size to the P/O ratio. Proc. Natl Acad. Sci. USA 107:16755–16756. 20858734 10.1073/pnas.1012260107 PMC2947903

[phy213007-bib-0019] Frick, N. T. , J. S. Bystriansky , Y. K. Ip , S. F. Chew , and J. S. Ballantyne . 2008 Lipid, ketone body and oxidative metabolism in the African lungfish, *Protopterus dolloi* following 60 days of fasting and aestivation. Comp. Biochem. Physiol. A Mol. Integr. Physiol. 151:93–101. 18598776 10.1016/j.cbpa.2008.06.004

[phy213007-bib-0020] Gnaiger, E. , G. Mendez , and S. C. Hand . 2000 High phosphorylation efficiency and depression of uncoupled respiration in mitochondria under hypoxia. Proc. Natl Acad. Sci. USA 97:11080–11085. 11005877 10.1073/pnas.97.20.11080 PMC27151

[phy213007-bib-0021] Guderley, H. , D. Lapointe , M. Bédard , and J.‐D. Dutil . 2003 Metabolic priorities during starvation: enzyme sparing in liver and white muscle of Atlantic cod, *Gadus morhua* L. Comp. Biochem. Physiol. A Mol. Integr. Physiol. 135:347–356. 12781835 10.1016/s1095-6433(03)00089-8

[phy213007-bib-0022] Hinkle, P. C. 2005 P/O ratios of mitochondrial oxidative phosphorylation. Biochim. Biophys. Acta 1706:1–11. 15620362 10.1016/j.bbabio.2004.09.004

[phy213007-bib-0023] Hung, S. S. O. , W. Liu , H. Li , T. Storebakken , and Y. Cui . 1997 Effect of starvation on some morphological and biochemical parameters in white sturgeon, *Acipenser transmontanus* . Aquaculture 151:357–363.

[phy213007-bib-0024] Jung, K. , and W. Henke . 1997 Effect of starvation on antioxidant enzymes and respiratory mitochondrial functions in kidney and liver from rats. J. Clin. Biochem. Nutr. 22:163–169.

[phy213007-bib-0025] Kadenbach, B. , R. Ramzan , and S. Vogt . 2013 High efficiency versus maximal performance — the cause of oxidative stress in eukaryotes: a hypothesis. Mitochondrion 13:1–6. 23178790 10.1016/j.mito.2012.11.005

[phy213007-bib-0026] Konstantinov, Y. M. , V. V. Lyakhovich , and A. V. Panov . 1976 A possible role of adenine nucleotide transport in the regulation of respiration of rat liver mitochondria. Bull. Exp. Biol. Med. 81:167–169. 1276407

[phy213007-bib-0027] Kuznetsov, A. V. , D. Strobl , E. Ruttmann , A. Konigsrainer , R. Margreiter , and E. Gnaiger . 2002 Evaluation of mitochondrial respiratory function in small biopsies of liver. Anal. Biochem. 305:186–194. 12054447 10.1006/abio.2002.5658

[phy213007-bib-0028] Layec, G. , A. Bringard , Y. Le Fur , J. P. Micallef , C. Vilmen , S. Perrey , et al. 2015 Opposite effects of hyperoxia on mitochondrial and contractile efficiency in human quadriceps muscles. Am. J. Physiol. Regul. Integr. Comp. Physiol. 308:R724–R733. 25695290 10.1152/ajpregu.00461.2014

[phy213007-bib-0029] Lehninger, A. L. , D. L. Nelson , and M. M. Cosx . 1993 Principles of biochemistry. 2nd ed. Worth Publishers, NY.

[phy213007-bib-0030] Leyssens, A. , A. V. Nowicky , L. Patterson , M. Crompton , and M. R. Duchen . 1996 The relationship between mitochondrial state, ATP hydrolysis, [Mg^2+^]i and [Ca^2+^]i studied in isolated rat cardiomyocytes. J. Physiol. 496:111–128. 8910200 10.1113/jphysiol.1996.sp021669 PMC1160828

[phy213007-bib-0031] Makrecka‐Kuka, M. , G. Krumschnabel , and E. Gnaiger . 2015 High‐resolution respirometry for simultaneous measurement of oxygen and hydrogen peroxide fluxes in permeabilized cells, tissue homogenate and isolated mitochondria. Biomolecules 5:1319–1338. 26131977 10.3390/biom5031319 PMC4598754

[phy213007-bib-0032] Monternier, P.‐A. , V. Marmillot , J.‐L. Rouanet , and D. Roussel . 2014 Mitochondrial phenotypic flexibility enhances energy savings during winter fast in king penguin chicks. J. Exp. Biol. 217:2691–2697. 24803465 10.1242/jeb.104505

[phy213007-bib-0033] Monternier, P. , A. Fongy , F. Hervant , J. Drai , D. Collin‐Chavagnac , J. Rouanet , et al. 2015 Skeletal muscle heterogeneity in fasting‐induced mitochondrial oxidative phosphorylation flexibility in cold‐acclimated ducklings. J. Exp. Biol. 218:2427–2434. 26026038 10.1242/jeb.122671

[phy213007-bib-0034] Ouhabi, R. , M. Boue‐Grabot , and J.‐P. Mazat . 1998 Mitochondrial ATP synthesis in permeabilized cells: assessment of the ATP/O values *in situ* . Anal. Biochem. 263:169–175. 9799528 10.1006/abio.1998.2776

[phy213007-bib-0035] Pecinova, A. , Z. Drahota , H. Nuskova , P. Pecina , and J. Houstek . 2011 Evaluation of basic mitochondrial functions using rat tissue homogenates. Mitochondrion 11:722–728. 21664301 10.1016/j.mito.2011.05.006

[phy213007-bib-0036] Pham, T. , D. Loiselle , A. Power , and A. J. R. Hickey . 2014 Mitochondrial inefficiencies and anoxic ATP hydrolysis capacities in diabetic rat heart. Am. J. Physiol. Cell. Physiol. 307:C499–C507. 24920675 10.1152/ajpcell.00006.2014

[phy213007-bib-0037] Postic, C. , R. Dentin , and J. Girard . 2004 Role of the liver in the control of carbohydrate and lipid homeostasis. Diabetes Metab. 30:398–408. 15671906 10.1016/s1262-3636(07)70133-7

[phy213007-bib-0038] Rovenko, B. M. , O. I. Kubrak , D. V. Gospodaryov , I. S. Yurkevych , A. Sanz , O. V. Lushchak , et al. 2015 Restriction of glucose and fructose causes mild oxidative stress independently of mitochondrial activity and reactive oxygen species in *Drosophila melanogaster* . Comp. Biochem. Physiol. A Mol. Integr. Physiol. 187:27–39. 25941153 10.1016/j.cbpa.2015.04.012

[phy213007-bib-0039] Salin, K. , L. Teulier , B. Rey , J. L. Rouanet , Y. Voituron , C. Duchamp , et al. 2010a Tissue variation of mitochondrial oxidative phosphorylation efficiency in cold‐acclimated ducklings. Acta Biochim. Pol. 57:409–412. 21125027

[phy213007-bib-0040] Salin, K. , Y. Voituron , J. Mourin , and F. Hervant . 2010b Cave colonization without fasting capacities: an example with the fish *Astyanax fasciatus mexicanus* . Comp. Biochem. Physiol. A Mol. Integr. Physiol. 156:451–457. 20382251 10.1016/j.cbpa.2010.03.030

[phy213007-bib-0041] Salin, K. , E. Luquet , B. Rey , D. Roussel , and Y. Voituron . 2012a Alteration of mitochondrial efficiency affects oxidative balance, development and growth in frog (*Rana temporaria*) tadpoles. J. Exp. Biol. 215:863–869. 22323209 10.1242/jeb.062745

[phy213007-bib-0042] Salin, K. , D. Roussel , B. Rey , and Y. Voituron . 2012b David and Goliath: a mitochondrial coupling problem? J. Exp. Zool. A Ecol. Genet. Physiol. 317:283–293. 25363578 10.1002/jez.1722

[phy213007-bib-0043] Salin, K. , S. K. Auer , B. Rey , C. Selman , and N. B. Metcalfe . 2015 Variation in the link between oxygen consumption and ATP production, and its relevance for animal performance. Proc. Biol. Sci. 282:20151028. 26203001 10.1098/rspb.2015.1028 PMC4528520

[phy213007-bib-0044] Salin, K. , S. K. Auer , G. J. Anderson , C. Selman , and N. B. Metcalfe . 2016 Inadequate food intake at high temperatures is related to depressed mitochondrial respiratory capacity. J. Exp. Biol. 219:1356–1362. 26944497 10.1242/jeb.133025

[phy213007-bib-0045] Savina, M. V. , L. V. Emel'yanova , S. M. Korotkov , I. V. Brailovskaya , and A. D. Nadeev . 2009 Bioenergetics of mitochondria of the liver with biliary atresia during prolonged starvation. Dokl. Biochem. Biophys. 425:80–83. 19496327 10.1134/s1607672909020069

[phy213007-bib-0046] Szmacinski, H. , and J. R. Lakowicz . 1996 Fluorescence lifetime characterization of magnesium probes: improvement of Mg^2+^ dynamic range and sensitivity using phase‐modulation fluorometry. J. Fluoresc. 6:83–95. 24227082 10.1007/BF00732047 PMC6897576

[phy213007-bib-0047] Theron, M. , F. Guerrero , and P. Sebert . 2000 Improvement in the efficiency of oxidative phosphorylation in the freshwater eel acclimated to 10.1 MPa hydrostatic pressure. J. Exp. Biol. 203:3019–3023. 10976038 10.1242/jeb.203.19.3019

[phy213007-bib-0048] Trzcionka, M. , K. W. Withers , M. Klingenspor , and M. Jastroch . 2008 The effects of fasting and cold exposure on metabolic rate and mitochondrial proton leak in liver and skeletal muscle of an amphibian, the cane toad *Bufo marinus* . J. Exp. Biol. 211:1911–1918. 18515721 10.1242/jeb.016519

[phy213007-bib-0049] Van Bergen, N. J. , R. E. Blake , J. G. Crowston , and I. A. Trounce . 2014 Oxidative phosphorylation measurement in cell lines and tissues. Mitochondrion 15:24–33. 24657935 10.1016/j.mito.2014.03.003

[phy213007-bib-0050] Watt, I. N. , M. G. Montgomery , M. J. Runswick , A. G. W. Leslie , and J. E. Walker . 2010 Bioenergetic cost of making an adenosine triphosphate molecule in animal mitochondria. Proc. Natl Acad. Sci. USA 107:16823–16827. 20847295 10.1073/pnas.1011099107 PMC2947889

[phy213007-bib-0051] Wu, M. , A. Neilson , A. L. Swift , R. Moran , J. Tamagnine , D. Parslow , et al. 2007 Multiparameter metabolic analysis reveals a close link between attenuated mitochondrial bioenergetic function and enhanced glycolysis dependency in human tumor cells. Am. J. Physiol. Cell. Physiol. 292:C125–C136. 16971499 10.1152/ajpcell.00247.2006

[phy213007-bib-0052] Ziak, J. , A. Krajcova , K. Jiroutkova , V. Nemcova , V. Dzupa , and F. Duska . 2015 Assessing the function of mitochondria in cytosolic context in human skeletal muscle: adopting high‐resolution respirometry to homogenate of needle biopsy tissue samples. Mitochondrion 21:106–112. 25701243 10.1016/j.mito.2015.02.002

